# Dual-Mass MEMS Gyroscope Parallel Denoising and Temperature Compensation Processing Based on WLMP and CS-SVR

**DOI:** 10.3390/mi11060586

**Published:** 2020-06-11

**Authors:** Longkang Chang, Huiliang Cao, Chong Shen

**Affiliations:** Science and Technology on Electronic Test & Measurement Laboratory, North University of China, Taiyuan 030051, China; 1706014442@st.nuc.edu.cn

**Keywords:** MEMS gyroscope, denoising, temperature drift compensation, variational mode decomposition, cuckoo search, support vector regression

## Abstract

For the sake of decreasing the effects of noise and temperature error on the measurement accuracy of micro-electro-mechanical system (MEMS) gyroscopes, a denoising and temperature drift compensation parallel model method based on wavelet transform and forward linear prediction (WFLP) and support vector regression based on the cuckoo search algorithm (CS-SVR) is proposed in this paper. First, variational mode decomposition (VMD) is proposed in this paper, which is aimed at dividing the output signal of the gyroscope into intrinsic mode functions (IMFs); then, the IMFs are classified into three features—drift, mixed, and pure noise features—by the sample entropy (SE) value. Second, a wavelet transform and forward linear prediction (WFLP) are combined to remove the noise from the mixed features. Meanwhile, the drift feature is compensated by support vector regression based on the cuckoo search algorithm (CS-SVR). Finally, through reconstruction, the final signal is obtained. Experimental results demonstrate that the VMD-SE-WFLP-CS-SVR method proposed in this paper can decrease noise and compensate the temperature error effectively (angular random walking value is optimized from 1.667°/√h to 0.0667°/√h and the bias stability is reduced from 30°/h to 4°/h). In terms of denoising, the performance of the WFLP algorithm is superior to the wavelet threshold and FLP, as it combines their advantages; furthermore, in terms of temperature compensation, the proposed CS-SVR algorithm uses the cuckoo search algorithm to find the optimal parameters of SVR, improving the accuracy of the model.

## 1. Introduction

Recently, micro-electro-mechanical system (MEMS) gyroscopes have been widely studied and employed in aviation, attitude controlling, and guidance, on account of their excellent precision, fast response, and low cost, among other advantages [1,2,3]. Every coin has two sides, however: the performance of a MEMS gyroscope degrades rapidly with changes in temperature, which can limit its application [4]. Therefore, suppressing MEMS gyroscope temperature errors is a key issue. In order to ameliorate these temperature characteristics, a lot of research has been carried out. To be able to achieve an acceptable signal-to-noise ratio, it is necessary to compensate for manufacturing imperfections. For instance, in ring-type and axisymmetric resonators, methods stemming from the ring dynamics [5] can be used to tune the resonators [6,7], and similar techniques can be employed for tuning quadrupole gyros [8] and tuning for [9].

At present, hardware compensation and software compensation are two mainstream methods for temperature compensation. In terms of hardware methods, the structure of the MEMS gyroscope is optimized or the circuit of the MEMS gyroscope is controlled to perform temperature compensation. For example, Cui proposed a novel temperature compensation method based on compensation of the driving modal vibration characteristics with variable temperature resistance [10]. Cao designed a pole-zero temperature compensation circuit to compensate for temperature drift [11]. In [12], the bandwidth characteristics of MEMS gyroscopes were studied, aiming to suppress the noise of the MEMS sensor. However, hardware compensation cannot meet the needs of a fast response, and the temperature control system greatly increases the volume, weight, and cost. Such a large volume and weight is considered a fatal limitation.

The other method is software compensation, which establishes a temperature drift model to acquire temperature compensation by studying the law between the output signal and temperature of MEMS gyroscopes accurately. The most commonly used methods in software compensation are signal filtering and temperature compensation, which have been successfully applied especially in MEMS gyroscopes. In [13], an improved empirical mode decomposition (EMD)-ensemble extreme learning machine (ELM) model was proposed to compensate for temperature drift. A radial basis function neural network-genetic algorithm-Kalman filter (RBF NN-GA-KF)-based model has been established to improve the accuracy of MEMS gyroscopes effectively [14].

The factors that affect the precision of a MEMS gyroscope are have two portions that need to be solved: one is to eliminate the influence of noise of high-frequency variation, and the other is to compensate for the drift reflecting the low frequency change. The most commonly used techniques are serial processing and parallel processing. Serial processing methods consist of first denoising the signal with a filter and then establishing the temperature error model to compensate for the drift, the efficiency of which is low. In this paper, parallel processing [15,16] is employed, which implies that the noise and drift in the gyroscope signal are handled synchronously. Empirical mode decomposition (EMD) [17,18] and wavelet decomposition [19] are a popular multi-scale analysis method. However, mode mixing occurs in EMD; therefore, ensemble empirical mode decomposition (EEMD) has been put forward [20,21,22]. EEMD still has great drawbacks, such as potentially producing spurious modes, additional post-processing work, and large computational burden. Here, we propose a variational mode decomposition [23] with sample entropy, which can effectively avoid modal aliasing and over-decomposition while accurately reflecting the characteristics of the signal.

Machine learning algorithms such as extreme learning machine (ELM) [24], random forest (RF) [25], and logistic regression [26] have become an important research issue and have been widely applied in a variety of fields [27]. In this paper, we choose support vector regression (SVR) to establish temperature drift models for MEMS gyroscopes. The SVR maps the original data to higher dimensions through a kernel function, and then trains using samples to learn and predict with high accuracy. Finding the appropriate values for parameters is extremely important. Therefore, the cuckoo search (CS) optimization algorithm is proposed to globally optimize the parameters of SVR, which can improve the convergence speed of the algorithm and avoid blindly selecting parameters of SVR.

In this paper, the denoising method and temperature error modeling are designed for MEMS gyroscopes, a wavelet transform and forward linear prediction (WFLP) algorithm is introduced for eliminating the noise of output signal, and the CS-SVR model is proposed for temperature error modeling. The rest of paper is structured as follows: An introduction to dual-mass MEMS gyroscopes is given in Section 2. Section 3 describes the denoising and drift compensation algorithm. In Section 4, the temperature experiments are carried out and a comparison among algorithms is discussed to verify the favorable performance of the WLP-CS-SVR method. Finally, our conclusions are clearly provided in Section 5.

## 2. Dual-Mass MEMS Gyroscope

### 2.1. The Structure of MEMS Gyroscopes

**Figure 1:**
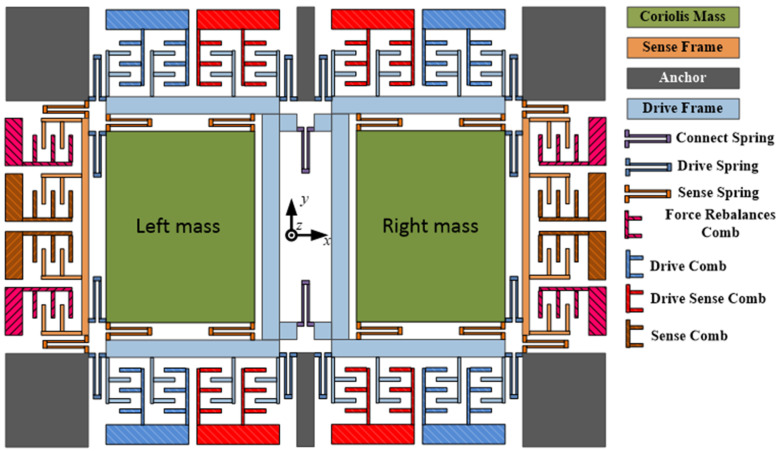
Schematic of the dual-mass gyro structure.

MEMS gyroscopes with a turning-fork structure are studied in this paper. Figure 1 shows the turning-fork gyroscope’s structure. The gyroscope structure contains two modes, a drive mode and a sensing mode, which are classified as follows: the drive mode contains three sections—drive springs, drive frame, and drive comb, and the sensing mode also contains three portions—sensing springs, sensing frame, and sensing comb. There is no coupling between the drive mode and sensor mode, but the mass is the part of both. In addition, when the drive mode is incurred by an electrostatic force, they vibrate in an opposite direction along the X-axis because they are merely able to move along the X-axis. Ω_z_, around in the Z-axis, is input following the angular rate and the Coriolis force is engendered by the vibrating quality, which is transferred into sense frame movement along the Y-axis; this movement is then detected by the monitor circuit.

**Figure 2:**
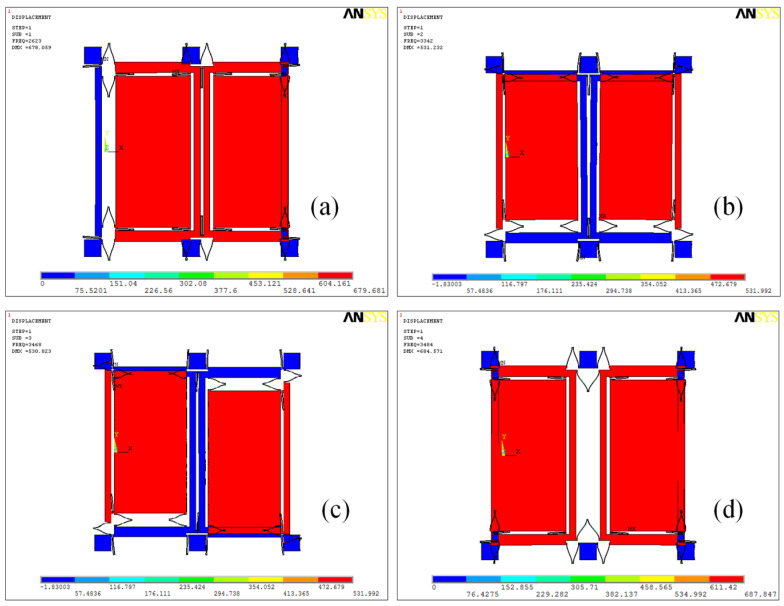
The first four order modes of dual-mass sensing mode-coupling micro-electro-mechanical system (MEMS) gyroscope structure: (**a**) drive in phase mode (first mode) with frequency ω_x1_ = 2623 × 2π rad/s; (**b**) sensing in phase mode (second mode) with frequency ω_y1_ = 3342 × 2π rad/s; (**c**) sensing anti-phase mode (third mode) with frequency ω_y2_ = 3468 × 2π rad/s; and (**d**) drive anti-phase mode (fourth mode) with frequency ω_x2_ = 3484 × 2π rad/s.

The drive mode in the gyroscope structure follows the turning-fork’s theory: the two drive masses are connected with the connect spring (with a U shape), and the two sense masses are connected with the drive spring. Figure 2 shows the first four order modes. In this paper, we used the Ansys software to mimic the system. From Figure 2, we can see that the frequency difference between the 1st mode and 4th mode is large (> 1000 Hz), while we know that the fourth mode’s quality factor is higher than 2000; therefore, the true working drive mode should be the fourth mode.

The first four order modes of the structure are analyzed and shown in Figure 2: Figure 2a shows the 1st mode (where the simulation resonant frequency is 2623 Hz), the drive in-phase mode, with the left and right masses moving in the same direction along the x-axis. Figure 2b shows the 2nd mode (where the simulation resonant frequency is 3342 Hz), the sensing in-phase mode, with left and right masses moving in the same direction along the y-axis. Figure 2c shows the 3rd mode (where the simulation resonant frequency is 3468 Hz), the sensing anti-phase mode, with left and right masses moving in opposite directions along the y-axis. Figure 2d shows the 4th mode (where the simulation resonant frequency is 3484 Hz), the drive anti-phase mode, with left and right masses moving in opposite directions along the x-axis. The drive anti-phase mode is the expected drive mode. Therefore, the left and right Coriolis masses both have two degrees of freedom (along the x- and y-axes), while the drive frame has one degrees of freedom (along the x-axis) and the sense frame has one degree of freedom (along the y-axis).

### 2.2. The Introduction of the Gyroscope Monitoring System 

**Figure 3:**
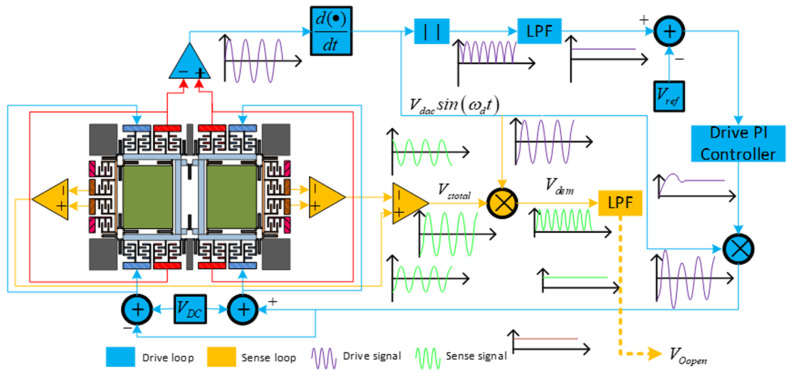
Gyroscope system schematic diagram.

As displayed in Figure 3, the displacement x(t) of the drive frame is measured by the drive sense combs. Then, the split amplifier was used to pick up the drive frame’s displacement. In order to meet the phase requirement of signal V_dac_Sin(w_d_t), the signal phase was delayed by 90°. After that, through a full-wave rectifier and a low-pass filter, V_dac_ was picked up and compared with the reference voltage V_ref_. Next, by utilizing the output of the comparator, the integrator controller generated a controlling signal, and a DC signal VDC was driven by the controlling signal to accumulate V_dac_Sin(w_d_t) to shape into an excitation to the drive mode. 

The right and left sense frame movement signals were detected with the differential detection amplifier, while a second differential amplifier processed the output signal, in order to generate the sensing mode movement signal V_stotal_. To acquire the sense mode movement signal, V_stotal_ was demodulated by V_dac_Sin(w_d_t) and, then, a low-pass filter was configured to eliminate the noise [28].

## 3. Algorithms

### 3.1. Variational Mode Decomposition (VMD)

Variational mode decomposition (VMD) is aimed to decompose an original input signal f(t) into a certain number of intrinsic mode functions (IMFs), u_k_, which are mostly compact and can be expressed as:

(1) $$u_{k}=A_{k}\cos(\varphi_{k}(t))$$

where A_k_(t) is the instantaneous amplitude of u_k_(t) and φ_k_(t) is the instantaneous phase.

Using the H1 Gauss smoothness of the demodulated signal, the bandwidth of u_k_(t) can be reckoned; this can be expressed as:

(2) $$\left\{ \begin{array}{l} \underset{\left(u_{k})(w_{k}\right)}{\min}\left\{\sum_{k}{\left\|\partial_{t}[\sigma (t)+\frac{j}{t}u_{t}(t)]e^{-jw_{k}t}\right\|}_{2}^{2}\right\}\\ s.t\sum_{k}u_{k}=f(t) \end{array}\right.$$

where f(t) is the original input signal, {u_k_} = {u_1_,u_2_,…,u_k_} are the k IMFs obtained by decomposing the signal, {w_k_} = {w_1_,w_2_,…,w_k_} stands for a series of center frequencies, and σ_t_ is the impulse function.

Generally, in order to solve a constrained variation problem, the Lagrange multiplier operator λ and the penalty factor α are used to transform the constrained variational problem into an unconstrained variational problem. With this, the augmented Lagrangian is described as follows:

(3) $$L(\left\{u_{k}\right\},\left\{w_{k}\right\},\lambda )=\alpha {\sum_{k=1}^{k}\left\|\partial_{t}\left[\left(\delta (t)+\frac{j}{\pi t}\right)\times u_{k}(t)\right]e^{iw_{k}t}\right\|}_{2}^{2}+{\left\|f(t)-\sum_{k=1}^{k}u_{k}(t)\right\|}_{2}^{2}+\langle \lambda (t),f(t)-\sum_{k=1}^{k}u_{k}(t)\rangle$$

where α is the balancing parameter and λ is the Lagrangian multiplier.

Through the above theoretical analysis of VMD, the time–frequency domain transform is performed, and the complete solution procedure can be obtained as follows:

(1) Let *n* = 0 and initialize some parameters, such as the modal number k, the balancing parameter α, the Lagrangian multiplier ${\hat{\lambda }}^{1}$, and {$u_{k}^{1}$} and {$x_{k}^{1}$}. Meanwhile, the corresponding center frequency $w_{k}^{1}$ should be initialized as zero.

(2) Let *n* = 1, start the loop, and update $u_{k}^{n+1}$, $w_{k}^{n+1}$, and k iteratively, according to Equations (4) and (5)

(4) $${\hat{u}}_{k}^{n+1}\left(\omega \right)=\frac{\hat{f}(\omega )-\sum_{i<k}{\overset{\wedge }{\underset{i}{u}}}^{n+1}(\omega )-\sum_{i<k}{\overset{\wedge }{\underset{i<k}{u}}}^{n+1}(\omega )+\frac{{\overset{\wedge }{\lambda }}^{n}(\omega )}{2}}{1+2\alpha {\left(\omega -\omega_{k}^{n}\right)}^{2}}$$

(5) $$\omega_{k}^{n+1}=\frac{\int_{0}^{\infty }\omega {\left|{\overset{\wedge }{\underset{k}{u}}}^{n+1}(\omega )\right|}^{2}d\omega }{\int_{0}^{\infty }{\left|{\overset{\wedge }{\underset{k}{u}}}^{n+1}(\omega )\right|}^{2}d\omega }$$

where ${\hat{u}}_{k}^{n+1}\left(w\right)$ and $\hat{\lambda }\left(w\right)\;$denote the Fourier transforms of $u_{k}^{n+1}$(w) and λ(w), respectively. 

(3) Update the Lagrangian multiplier λ_n+1_ in light of the following equation.

(6) $$\lambda^{n+1}(\omega )=\lambda^{n}(\omega )+\tau (f(\omega )-\sum_{k}^{n+1}u_{k}(w))$$

where *τ* is an update parameter and the Lagrangian multiplier is effectively shut-off by keeping its value at zero.

(4) Let *n* = *n*+1 and repeat step 2) until the convergence condition is satisfied; namely,

(7) $${\sum_{k}{\left\|u_{k}^{n+1}-u_{k}^{n}\right\|}_{2}^{2}/\left\|u_{k}^{n}\right\|}_{2}^{2}<\gamma$$

where γ > 0 is the discriminating accuracy.

Finally, k intrinsic mode functions have been obtained, and the decomposition is completed.

### 3.2. Sample Entropy (SE)

After the decomposition of VMD, the original signal was decomposed into 6 IMFs. This paper introduced sample entropy (SE) to divide the IMFs into different features and, then, to recombine the IMFs into a new subseries based on their approximate SE values, greatly reducing the computational complexity. The SE [29] was initially proposed by Richman for gauging the complexity of a time series. Therefore, a more complex sample sequence represents a larger entropy of the sample.

The calculation process of the SE, which can be obtained in five steps, is briefly introduced as follows:

Step 1: Suppose that the original data is {X_i_}={x_1_,x_2_,…,x_N_} with a length of N. Given the pre-embedded dimension *m* and similar tolerance *r*, an m-dimensional vector can be reconstructed as X(i) = [x_i_,x_i+1_,…,x_i+m-1_].

Step 2: The distance d_m_(X_i_,X_j_) between x(i) and x(j) is defined as the maximum value of the discrepancy between the corresponding individuals, as follows:

(8) $$d_{m}(X_{i},X_{j})=\underset{k}{\max}(\left|x_{i+k}-x_{j+k}\right|)\;,k=0\sim m-1$$

Step 3: The number of d_m_(X_i_,X_j_) less than *r* are counted and the ratio of this number to the total distance (N-m-1) is recorded as $B_{i}^{m}$(r):

(9) $$B_{i}^{m}(r)=\frac{1}{N-m}num\left\{d_{m}(X_{i},X_{j})<r\right\}$$

Step 4: Calculate the mean and define B^m^(r):

(10) $$B^{m}(r)=\frac{1}{N-m+1}\sum_{i=1}^{N-m+1}B_{i}^{m}(r)$$

Step 5: Expand the dimension to m+1, repeat Steps 1–4 to get B^m+1^(r), and further to get B^m+1^(r), which is defined as follows:

(11) $$B^{m+1}(r)=\frac{1}{N-m}\sum_{i=1}^{N-m}B_{i}^{m+1}(r)$$

Theoretically, the SE of the original sequence is defined as:

(12) $$SE(m,r)=\underset{N\to \infty }{\lim}\left\{-\ln\left(\frac{B^{m+1}(r)}{B^{m}(r)}\right)\right\}$$

When N is a finite number, the above equation can be expressed as:

(13) $$SE(m,r,N)=-\ln\left(\frac{B^{m+1}(r)}{B^{m}(r)}\right)$$

The SE value be decided by *m* and *r*. The SE corresponding to different embedding dimensions *m* and similar tolerances *r* is also different. In general, the value of SE calculated with m = 1 or 2 and r = 0.1–0.25 SD has reasonable statistical characteristics (where SD is the standard deviation of the original series).

### 3.3. Wavelet Transform and Forward Linear Prediction Algorithm (WFLP)

A wavelet transform is an excellent method to analyze and process signal time–frequency characteristics; readers interested in its theoretical analysis can refer to [19]. The main idea of FLP [30] is that the previous gyro signal is multiplied by the corresponding weight to forecast the gyro signal at the current moment. A brief summary is given as follows:

The estimated value of the current gyroscope signal is

(14) $$\hat{x}(n)=\sum_{p=1}^{N}c_{p}x(n-p)=C^{T}X(n-1)$$

where *X*(*n*−1) = {*x*(*n*−1),*x*(*n*−2),…,*x*(*n*−N)}*^T^*, *c_p_* are the tap-weights, and N is the order; the larger the value of N, the better of the filtering effect, but this will increase the calculation amount required for filtering.

According to the minimum mean square theory, the iterative adjustment equation for the weights can be obtained as follows:

(15) C(n+1)=C(n)+ηE[e(n)X(n−1)]

where *e*(*n*) = *x*(*n*) − *x*^(*n*) is the forward prediction error and *η* is a small value, which is used to control the convergence rate of the entire iteration process.

In the FLP prediction process, the prediction error is closely related to the step size. At the initial stage, the prediction error is large, such that a relatively large step size can be selected. When the prediction error has fallen to a certain level, a small step size is taken, in order to increase the accuracy of the output. Therefore, the value of *η* is calculated as follows:

(16) $$\eta (n)=\beta (1-\exp(-\alpha {\left|c\tan e(n)\right|}^{2}))$$

In this paper, a wavelet transform and FLP are combined, to improve the accuracy of the filter effectively. In general, the steps of the WFLP algorithm are implemented as follows:

(1) With regard to the mixed features, wavelet decomposition is performed, a wavelet basis function is chosen, and the number of decomposed layers can be ascertained. 

(2) The wavelet coefficients are processed and the FLP algorithm is used to denoise by forming an approximation coefficient as the input signal.

(3) Wavelet reconstruction is carried out. The final signal can be reconstructed from approximation coefficients (after FLP) and detail coefficients using the selected wavelet basis function.

### 3.4. CS-SVR Algorithm

In this section, we first reviewed the theory of support vector regression (SVR) and then introduced the cuckoo search (CS), which is employed to optimize the parameters of SVR.

The main idea of SVR is to predict a desired value as output by looking for a regression function based on a series of input data. In order to construct the optimal decision function, the kernel function of the original space is used to replace the operation of dot product in the high-dimensional space.

The expression of the SVR algorithm is shown as follows:

(17) $$y_{i}=f(x_{i})=w^{T}\phi (x_{i})+b,\;i=1,2,\ldots ,N$$

where *w* denotes the weight factor of the SVR, *Φ* is the non-linear function, which projects the *x_i_* to high-dimensional space, and *b* is the threshold. 

SVR uses the insensitive loss function of ε-SVR to perform linear regression in the feature space, and transforms the regression problem into a convex quadratic programming problem with respect to the variables *w* and *b*. The SVR optimization problem is shown as Equation (18).

(18) $$\underset{w,b}{\min}P=\frac{1}{2}w^{T}w+C\sum_{i=1}^{l}\left(\xi_{i}+\xi_{i}^{*}\right)$$

(19) $$\mathrm{subject}\;\mathrm{to}\;\left\{ \begin{array}{l} y_{i}-w^{T}\phi (x)-b\leq \varepsilon +\xi_{i}\\ w^{T}\phi (x)+b-y_{i}\leq \varepsilon +\xi_{i}^{*}\\ \xi_{i},\xi_{i}^{*}\geq 0,i=1,2,\ldots l \end{array}\right.$$

where *ξ_i_* and $\xi_{i}^{*}$ are relaxation factors and *C* is a regularization parameter, which is used to control the degree of penalty for samples that exceed the error.

The kernel function *K*(*x_i_,x_j_*) = *Φ*(*x_i_*) × *Φ*(*x_j_*) is introduced by using the duality principle and Lagrange function. Common kernel functions include polynomial kernel functions, linear kernel functions, and radial basis kernel functions. In this paper, we selected the radial basis kernel function, due to its strong flexibility, wide range of use, and high regression accuracy; its expression is

(20) $$K(x_{i},x_{j})=\exp(-{\left\|x_{i}-x_{j}\right\|}^{2}/2\sigma^{2})$$

where σ is the kernel function parameter.

Then, the dual problem can be constructed, which is written as Equation (21)

(21) $$\underset{\alpha ,\alpha^{*}}{\min}D=\frac{1}{2}\sum_{i=1}^{l}\sum_{j=1}^{l}K(x_{i},x_{j})(\alpha_{i}-\alpha_{i}^{*})(\alpha_{j}-\alpha_{j}^{*})-\sum_{i=1}^{l}y_{i}(\alpha_{i}-\alpha_{i}^{*})+\sum_{i=1}^{l}\varepsilon (\alpha_{i}+\alpha_{i}^{*})$$

(22) $$\mathrm{subject}\;\mathrm{to}\;\left\{ \begin{array}{l} \alpha_{i},\alpha_{i}^{*}\in [0,C],\;i=1,2,\ldots ,l\\ \sum_{i=1}^{l}\left(\alpha_{i}-\alpha_{i}^{*}\right)=0 \end{array}\right.$$

where *α_i_* and $\alpha_{i}^{*}$ are Lagrange multipliers.

Finally, we could obtain the final regression function by solving the values of *α_i_* and $\alpha_{i}^{*}$, as follows:

(23) $$y=f(x)=\sum_{i=1}^{l}\left(\alpha_{i}-\alpha_{i}^{*}\right)\times \exp(-\left\|x-x_{i}\right\|/2\sigma^{2})+b$$

It is well-known that the regularization parameter C and the kernel function parameter σ are related to the prediction performance of SVR. It is important to choose appropriate parameters, as they can improve the generalization ability and prediction accuracy of SVR. Therefore, we used the CS algorithm to optimize the SVR parameters in this paper.

The cuckoo search algorithm is a naturally inspired search algorithm, which was developed based on the cuckoo’s nest-parasitic breeding strategy and the strategy of Levy flight. It focuses on three regulations, which are assumed:

Each egg is laid by one cuckoo once and the egg will be casually put into an unknown nest;

Nests with optimal egg production quality are prepared to be passed on to the next generation;

The number of available host nests is fixed and the probability of host bird discovering cuckoo eggs is P_α_ ∈ [0, 1]. Under this circumstance, they will throw the eggs away or give up the original nest and then establish a new nest.

Therefore, the equation for cuckoo patrol for nests and location update is shown as follows:

(24) $$e_{i}^{\left(t+1\right)}=e_{i}^{\left(t\right)}+\beta ⊕K(\lambda )\;i=1,2,\ldots ,n$$

where $e_{i}^{\left(t\right)}$ and $e_{i}^{\left(t+1\right)}$ are the position of generation *t* and *t+1* for the *i*th cuckoo’s nest, β is used to control the step size, ⊕ denotes point-to-point multiplication, and K(λ) is the path of random search.

**Figure 4:**
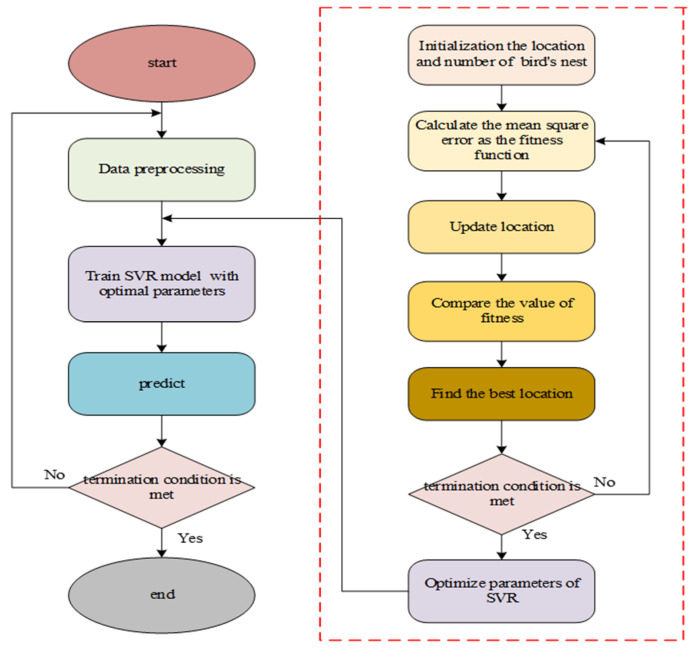
Flow chart of the support vector regression based on the cuckoo search (CS-SVR) algorithm.

The process of this method is displayed in Figure 4. From Figure 4, we can see that it uses the fitness function to find the optimal bird nest position, after which Levy flight is utilized to renew the position of the nest, meanwhile a novel nest and the previous generation’s fitness value are determined. Finally, the optimal bird’s nest position is found, which is the optimal value of the parameters of SVR.

### 3.5. Proposed VMD-SE-WFLP-CS-SVR Algorithm

**Figure 5:**
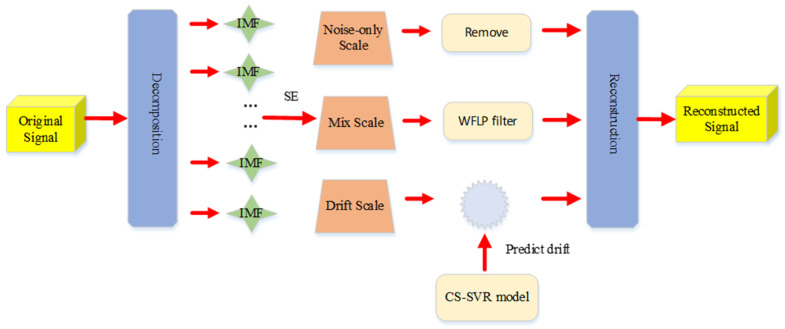
Flow chart of the variational mode decomposition (VMD)-sample entropy (SE)-wavelet transform and forward linear prediction (WFLP)-CS-SVR algorithm.

Figure 5 is the processing method for a gyroscope signal. The steps of denoising and temperature compensation are as follows:

(1) The original signal is first resolved into six components by VMD, then the SE value per component is computed to dividing the components into three features: drift, mixed (which is a combination of drift and noise), and noise-only features.

(2) The drift, mixed, and noise-only features are processed respectively; that is, the noise-only feature is directly removed, the mixed feature is filtered by the WFLP algorithm and the drift feature is compensated, based on the CS-SVR method.

(3) By denoising and compensation, the final signal is reconstructed.

## 4. Experiments and Comparisons

### 4.1. Experiments

**Figure 6:**
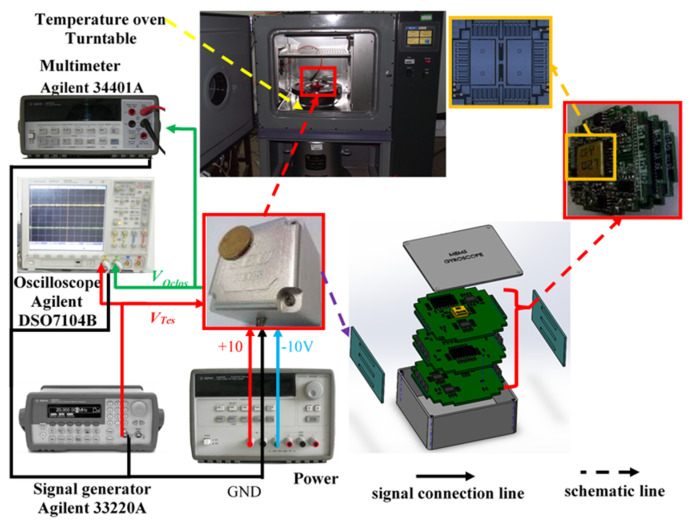
Experimental device.

For the sake of verifying the feasibility and effectiveness of the VMD-SE-WFLP-CS-CVR method, gyroscope temperature characteristics were tested through a temperature experiment. The gyroscope and the experimental equipment are displayed in Figure 6. A detection circuit was arranged and metal pins were not only used in linking their electronic signal but also the structures in each printed circuit board (PCB). The three PCBs are wrapped with a rubber pad (which we chose), and then were put into a metal shell, which could effectively preserve not only the structural chip but also the PCB against impact. In addition, in order to shield the electromagnetic field, the metal shell and the grounding signal were linked together. As mentioned above, there were three PCBs: the first PCB was linked to the structure chip and also used as an interface to process weak signals. The remaining two PCBs were also indispensable, which were the sense and drive circuits, respectively.

The experimental setup was equipped with a temperature control oven, which was used to create different temperature environments; a multimeter (Agilent 34401A, Agilent, Santa Clara, CA, USA), which accurately collected the output signal of the MEMS gyroscope; a signal generator (Agilent 33220A), which was used to generate a test voltage; and an Agilent E3631A DC Power Supply, used to provide ±10 V DC voltage and ground (GND).

**Figure 7:**
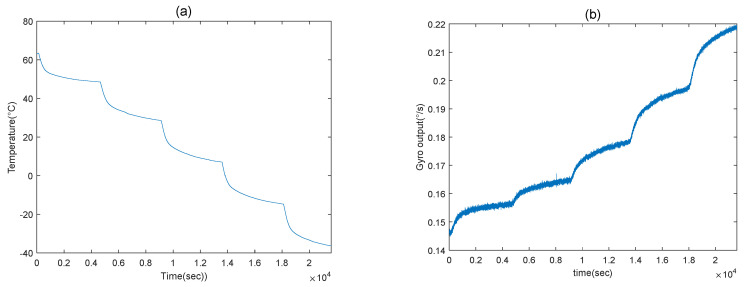
The results of temperature experiments.

The experimental process was as follows: Firstly, under room temperature, the gyroscope was kept in the state of being turned on for an hour. Then, the oven temperature was set to 60 °C quickly by warming up. After that, the oven was forced to hold at this temperature for an hour, in order to guarantee that the temperature inside the gyroscope shell reached 60 °C. Meanwhile, in order to ensure that the temperature of the inner shell of the gyroscope was not only steady but also kept with that of the oven, when the temperature of the oven dropped by 10 °C, it was stopped for one hour. Finally, when the oven temperature reached −40 °C, it was held for the last hour, and then the test was finished. Figure 7 shows the experimental results, from which we could conclude that, when the temperature changes obviously, so does the output of the MEMS gyroscope. Consequently, we were obliged to build the model for temperature compensation, which can eliminate the drift effectively.

### 4.2. Experimental Results

**Figure 8:**
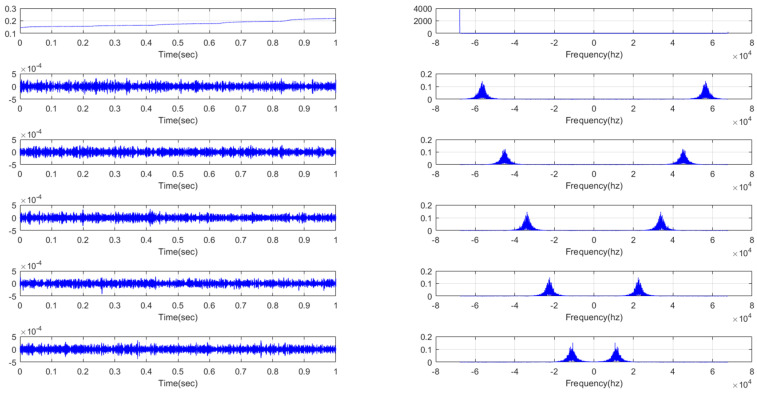
The diagram of VMD decomposition.

The result of the VMD decomposition diagram is shown in Figure 8, from which we can clearly see that the output signal of the gyroscope was decomposed into six IMFs with different frequencies. There is a huge amount of calculation required and the accumulated error of each decomposed component was processed to reconstruct the final signal. Therefore, after VMD decomposition was finished, the SE algorithm was employed to compute the SE values of each IMF.

**Figure 9:**
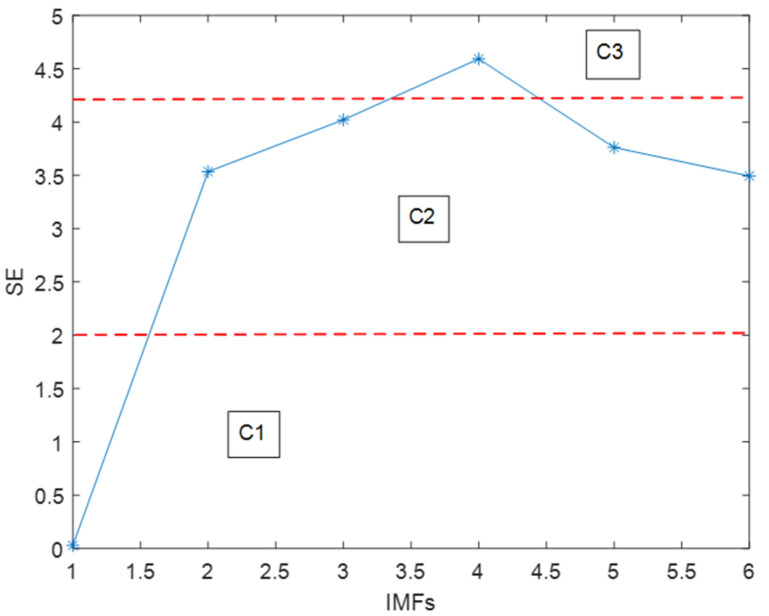
The SEs value corresponding to each intrinsic mode function (IMF).

**Figure 10:**
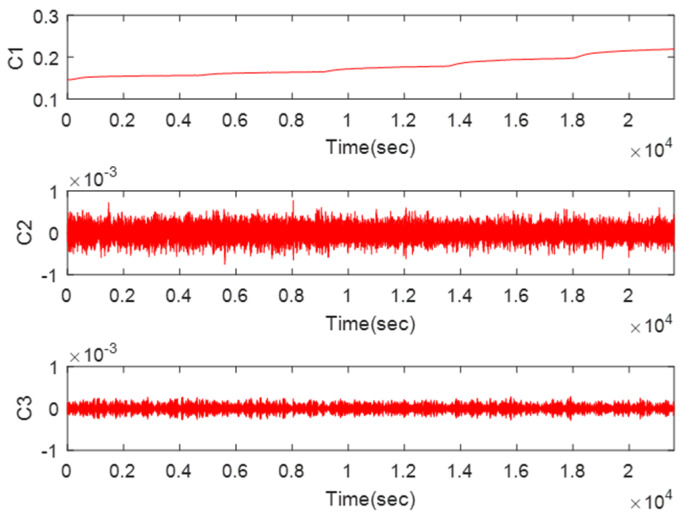
Extracting feature scales by SE.

Figure 9 shows the calculation results of SE. The SE can represent the randomness of a time series: the larger the value, the closer the time series is to random. We could classify the six IMFs into three groups by their respective SE values. According to the summary of the experiment, when the SE value was bigger than 4.2, the IMF was nearly completely noise; which has nothing to do with the reconstructed signal. When SE values are between 4.2 and 2, those IMFs are considered as mixed features, which contain both noise and useful signal. When the value is lower than 2, this IMF is a drift feature. From Figure 9, this means the 1st IMF is a drift feature (C1), the 2nd, 3rd, 5th, and 6th are mixed features (C2) and the 4th is a noise-only feature (C3). Figure 10 displays the results when the three features are recombined, respectively.

**Figure 11:**
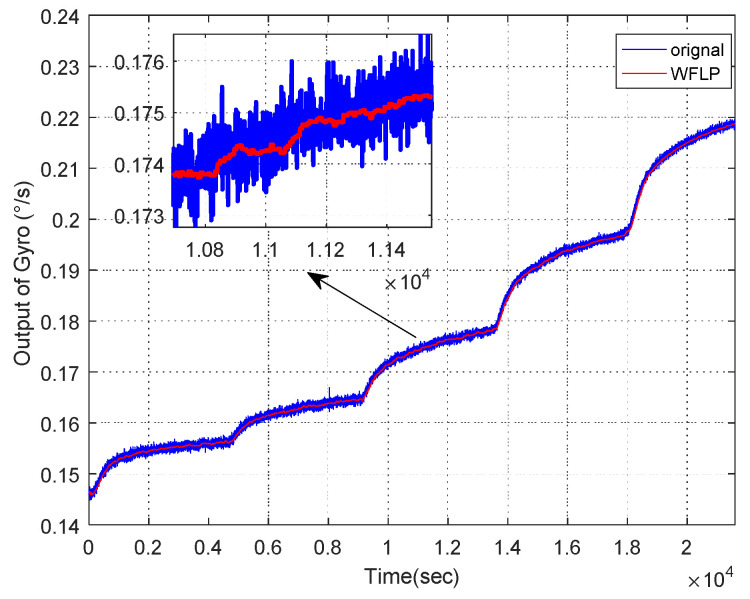
Original signal compared with WFLP.

**Figure 12:**
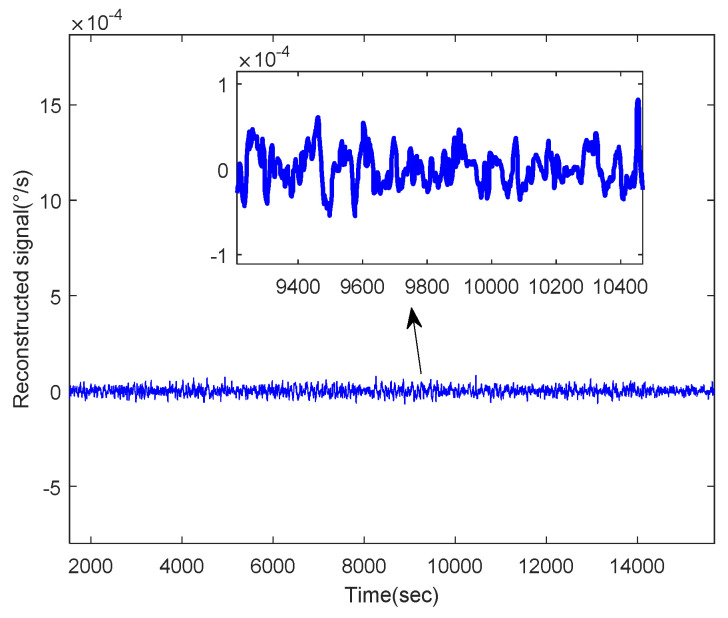
The final reconstructed signal.

In the first place, C1 was wiped off directly and C2 was filtered by the WFLP algorithm. Then, Figure 11 shows the reconstructed signal based on C2, which was denoised and C3, which was not processed. From Figure 11, we can see that the VMD-SE-WFLP decomposition method has good denoising performance. Finally, C3 was compensated for by the established CS-SVR model, and Figure 12 shows the final reconstructed signal, which was obtained after C2 was filtered and C3 was compensated for. We could conclude that the proposed VMD-SE-WFLP-CS-SVM parallel processing model could eliminate the influence of noise and drift in a MEMS gyroscope effectively. Comparisons among methods were given in the next section, to demonstrate its good performance.

### 4.3. Comparisons 

In this section, we first utilized the Allan variance for testing the superiority of the proposed method. Then, we compared WFLP with wavelet threshold and FLP, to prove that the WFLP algorithm had a better denoising effect. Finally, we analyzed the advantages of CS-SVR, where the prediction results demonstrated that CS-SVR could improve the prediction precision by finding the best SVR parameters.

#### 4.3.1. Comparison Between Original Signal and Processed Signal

**Figure 13:**
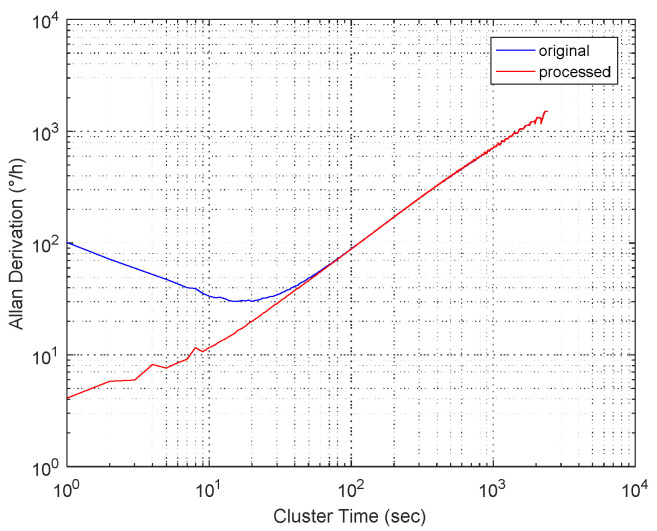
Allan variance analysis.

For the sake of verifying the effectiveness and accuracy of the proposed algorithm, Allan variance was employed. Allen variance has been widely used in the analysis and modeling of the gyroscope random error [31]. Through a series of operations, Figure 13 displays the Allan variance of the MEMS gyroscope’s original signal and the processed signal, based on the WFLP-CS-SVR parallel model. From Figure 13, we can see that the original signal had an angular random walking value, which is 1.667°/√h, and the same value of the signal after processing was 0.0667°/√h, indicating obvious optimization. The bias stability of the original signal was 30°/h, while the bias stability of the processed signal was 4°/h. Therefore, by the Allan analysis, we could clearly see that the WFLP-CS-SVR parallel model had great performance to compensate for temperature error in MEMS gyroscopes.

#### 4.3.2. Comparison Among Wavelet Threshold, FLP, and WFLP

**Figure 14:**
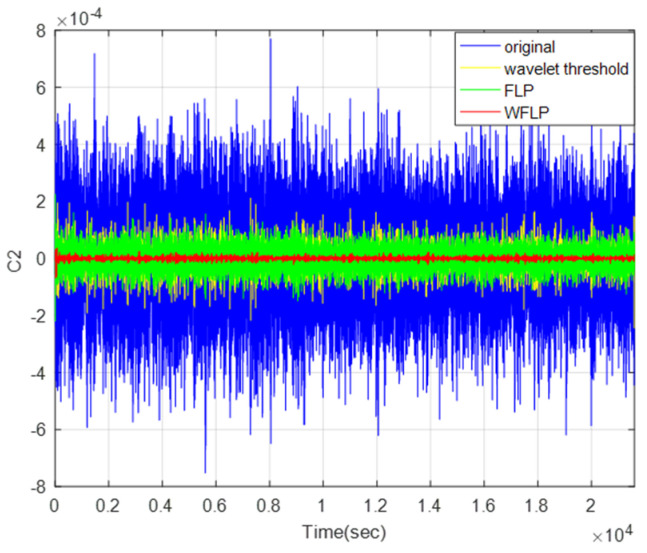
Comparison of denoising effects in wavelet threshold, FLP, and WFLP.

**Figure 15:**
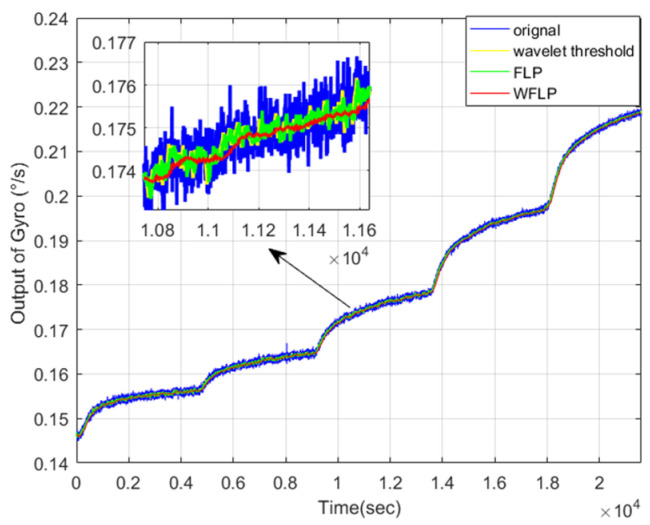
Reconstructed signal after denoising.

From the above analysis, the mixed feature C2 needs to be denoised. Therefore, we compared the denoising effects of the wavelet threshold, FLP, and WFLP, as displayed in Figure 14. We could see that the WFLP algorithm had the best performance, in terms of denoising. The WFLP algorithm makes the most of the superiority of the wavelet transform in signal multi-scale decomposition and the high-precision denoising of the FLP algorithm, which effectively reduces the impact of noise on the output of MEMS gyroscope, on the basis of only adding a small amount of complexity. Finally, the signal after reconstruction was exhibited in Figure 15, which shows that WFLP was more effective in eliminating the MEMS gyroscope noise.

#### 4.3.3. Comparison Between SVR and CS-SVR

**Figure 16:**
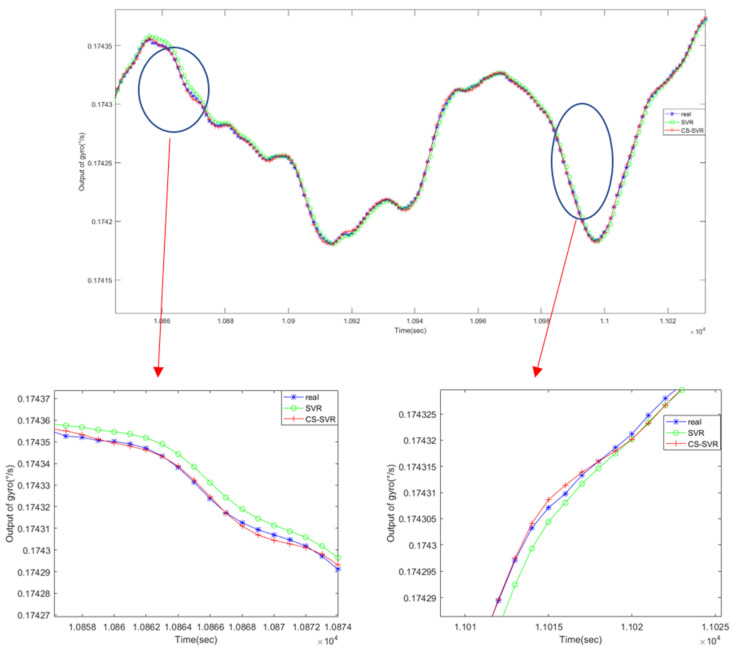
Comparison of the prediction results between SVR and CS-SVR.

Figure 16 shows the comparison between SVR and CS-SVR. For the sake of reflecting the superiority and universality of the CS-SVR algorithm, a group of original outputs of gyroscopes were chosen to train the model, then we stochastically chose a portion and magnified it, as shown in the Figure below. From Figure 16, we can see articulately that the result predicted by CS-SVR was closer to the real value. Through analysis, we know that, during the operation of the vector regression machine, there will be shortcomings of parameter optimization and prone to being trapped into local optimum, such that the SVR prediction results are relatively inaccurate. Therefore, the CS algorithm, with both global and strong local search ability, was chosen to optimize the model parameters, effectively solving this problem. Therefore, comparing the SVR algorithm, CS-SVR has better accuracy prediction and effect for the temperature compensation modeling of MEMS gyroscopes.

## 5. Conclusions

In this paper, we put forward a denoising and temperature compensation method based on VMD-SE, WFLP, and CS-SVR for MEMS gyroscopes. First of all, the parallel model was able to restrain the influence of noise very well, while the drift feature was extracted exactly, helping to establish a highly accurate compensation model. As the temperature errors were strongly non-stationary, the SE algorithm was used to classify the original signal into drift, mixed, and pure noise features, greatly decreasing the cost of calculation. Then, considering the aspect of denoising, the WFLP was capable of dispelling noise better, compared to the wavelet threshold and FLP methods alone. For compensating the drift of temperature, CS-SVR had higher accuracy and precision than the traditional SVR algorithm. Temperature experiments were carried out, and the results demonstrated that, in the temperature range from −40 to 60 °C, the angular random walk and the bias stability of the processed signal are decreased by 96% and 87%, compared with the original signal, when using the proposed method. The results indicate that the VMD-SE-WFLP-CS-SVR method proposed in this paper has excellent accuracy and effect.
